# Notification that new names of prokaryotes, new combinations and new taxonomic opinions have appeared in volume 75, part 6 of the IJSEM

**DOI:** 10.1099/ijsem.0.006862

**Published:** 2025-10-10

**Authors:** Aharon Oren, Markus Göker

**Affiliations:** 1 The Institute of Life Sciences, The Hebrew University of Jerusalem, The Edmond J. Safra Campus, 9190401 Jerusalem, Israel; 2 Leibniz Institute DSMZ – German Collection of Microorganisms and Cell Cultures, Inhoffenstrasse 7B, 38124 Braunschweig, Germany

This listing of names of prokaryotes published in a previous issue of the *IJSEM* is provided as a service to microbiology to assist in the recognition of new names and new combinations. This procedure was proposed by the Judicial Commission [1]. The names given herein are listed according to the rules of priority (i.e. time and date of publication of the articles on the journal’s platform and the order of valid publication of names in the original articles) [2].

**Table:** 

Order of article publication	Name/authors	Proposed as	Article no.	Page no.
1	*Pedobacter anseongensis* Jo *et al*. 2025	sp. nov.	006800	9
1	*Pedobacter immunditicola* Jo *et al*. 2025	sp. nov.	006800	11
1	*Pedobacter superstes* Jo *et al*. 2025	sp. nov.	006800	11
2	*Faecalibacterium langellae* Ang *et al*. 2025∗	sp. nov.	006804	6
3	*Roseovarius roseus* Yang *et al*. 2025	sp. nov.	006782	6
3	*Roseovarius maritimus* Yang *et al*. 2025	sp. nov.	006782	8
4	*Gaoshiqia hydrogeniformans* Ueno *et al*. 2025	sp. nov.	006802	9
5	*Pseudolactococcus* Abe *et al*. 2025	gen. nov.	006803	6
5	*Pseudolactococcus yaeyamensis* Abe *et al*. 2025	sp. nov.	006803	6
5	*Pseudolactococcus carnosus* (Hilgarth *et al*. 2020) Abe *et al*. 2025	comb. nov. [basonym: *Lactococcus carnosus* Hilgarth *et al*. 2020]	006803	10
5	*Pseudolactococcus chungangensis* (Cho *et al*. 2008) Abe *et al*. 2025	comb. nov. [basonym: *Lactococcus chungangensis* Cho *et al*. 2008]	006803	11
5	*Pseudolactococcus hodotermopsidis* (Noda *et al*. 2020) Abe *et al*. 2025	comb. nov. [basonym: *Lactococcus hodotermopsidis* Noda *et al*. 2020]	006803	11
5	*Pseudolactococcus insecticola* (Noda *et al*. 2020) Abe *et al*. 2025	comb. nov. [basonym: *Lactococcus insecticola* Noda *et al*. 2020]	006803	11
5	*Pseudolactococcus laudensis* (Meucci *et al*. 2015) Abe *et al*. 2025	comb. nov. [basonym: *Lactococcus laudensis* Meucci *et al*. 2015]	006803	11
5	*Pseudolactococcus paracarnosus* (Hilgarth *et al*. 2020) Abe *et al*. 2025	comb. nov. [basonym: *Lactococcus paracarnosus* Hilgarth *et al*. 2020]	006803	11
5	*Pseudolactococcus piscium* (Williams *et al*. 1990) Abe *et al*. 2025	comb. nov. [basonym: *Lactococcus piscium* Williams *et al*. 1990]	006803	12
5	*Pseudolactococcus plantarum* (Collins *et al*. 1984) Abe *et al*. 2025	comb. nov. [basonym: *Streptococcus plantarum* Collins *et al*. 1984]	006803	12
5	*Pseudolactococcus raffinolactis* (Orla-Jensen and Hansen 1932) Abe *et al*. 2025	comb. nov. [basonym: *Streptococcus raffinolactis* Orla-Jensen and Hansen 1932 (Approved Lists 1980)]	006803	12
5	*Pseudolactococcus reticulitermitis* (Yuki *et al*. 2018) Abe *et al*. 2025	comb. nov. [basonym: *Lactococcus reticulitermitis* Yuki *et al*. 2018]	006803	12
5	*Lactovum odontotermitis* Abe *et al*. 2025	sp. nov.	006803	13
6	*Chryseobacterium cupriresistens* Arroyo-Herrera *et al*. 2025	sp. nov.	006806	7
7	*Methylobacterium oryzisoli* Liu *et al*. 2025	sp. nov.	006805	8
8	*Pseudomonas lyxosi* Zhao *et al*. 2025	sp. nov.	006799	4
8	*Pseudomonas arabinosi* Zhao *et al*. 2025	sp. nov.	006799	5
8	*Pseudomonas frigoris* Zhao *et al*. 2025	sp. nov.	006799	6
9	*Streptomyces sediminimaris* Emthomya *et al*. 2025	sp. nov.	006811	9
10	*Amycolatopsis ponsaeliensis* Thompson *et al*. 2025	sp. nov.	006810	11
11	*Georgenia wangjunii* Ma *et al*. 2025	sp. nov.	006762	6
11	*Georgenia sunbinii* Ma *et al*. 2025	sp. nov.	006762	7
12	*Flavobacterium mekongense* Phithakrotchanakoon *et al*. 2025	sp. nov.	006815	13
13	*Antiquaquibacter soli* (Wan *et al*. 2024) Gtari *et al*. 2025	comb. nov. [basonym: *Salinibacterium soli* Wan *et al*. 2024]	006809	8
13	*Antiquaquibacter metalliresistens* (Liu *et al*. 2024) Gtari *et al*. 2025	comb. nov. [basonym: *Salinibacterium metalliresistens* Liu *et al*. 2024]	006809	8
13	*Homoserinimonas hongtaonis* (Li *et al*. 2019) Gtari *et al*. 2025	comb. nov. [basonym: *Salinibacterium hongtaonis* Li *et al*. 2019]	006809	9
13	*Homoserinimonas sedimenticola* (Lu *et al*. 2024) Gtari *et al*. 2025	comb. nov. [basonym: *Salinibacterium sedimenticola* Lu *et al*. 2024]	006809	9
13	*Salinibacterium rubrum* (Reddy *et al*. 2003) Gtari *et al*. 2025	comb. nov. [basonym: *Leifsonia rubra* Reddy *et al*. 2003]	006809	9
13	*Leifsonia tongyongensis* (Kim *et al*. 2014) Gtari *et al*. 2025	comb. nov. [basonym: *Diaminobutyricibacter tongyongensis* Kim *et al*. 2014]	006809	9
13	*Glaciibacter psychrotolerans* (Ganzert *et al*. 2011) Gtari *et al*. 2025	comb. nov. [basonym: *Leifsonia psychrotolerans* Ganzert *et al*. 2011]	006809	10
13	*Glaciibacter kafniensis* (Pindi *et al*. 2009) Gtari *et al*. 2025	comb. nov. [basonym: *Leifsonia kafniensis* Pindi *et al*. 2009]	006809	10
13	*Leifsonella* Gtari *et al*. 2025	gen. nov.	006809	10
13	*Leifsonella bigeumensis* (Dastager *et al*. 2008) Gtari *et al*. 2025	comb. nov. [basonym: *Leifsonia bigeumensis* Dastager *et al*. 2008]	006809	10
13	*Orlajensenia* Gtari *et al*. 2025	gen. nov.	006809	10
13	*Orlajensenia flava* (An *et al*. 2023) Gtari *et al*. 2025	comb. nov. [basonym: *Glaciibacter flavus* An *et al*. 2023]	006809	11
13	*Orlajensenia leifsoniae* Gtari *et al*. 2025	sp. nov.†	006809	11
14	*Lacticaseibacillus salsurae* Song *et al*. 2025	sp. nov.	006816	9
14	*Levilactobacillus muriae* Song *et al*. 2025	sp. nov.	006816	11
15	*Arcobacter iocasae* Zhang *et al*. 2025	sp. nov.	006818	5
16	*Bradyrhizobium tunisiense* Hsouna *et al*. 2025	sp. nov.	006807	9
17	*Microbacterium jiangjiandongii* Ye *et al*. 2025	sp. nov.	006821	7
17	*Microbacterium wangruii* Ye *et al*. 2025	sp. nov.	006821	8
18	*Faucicola osloensis* (Bøvre and Henriksen 1967) Sbissi *et al*. 2025‡	comb. nov. [basonym: *Moraxella osloensis* Bøvre and Henriksen 1967 (Approved Lists 1980)]	006820	9
	*Faucicola atlantae* (Bøvre *et al*. 1976) Sbissi *et al*. 2025‡	comb. nov. [basonym: *Moraxella atlantae* Bøvre *et al*. 1976 (Approved Lists 1980)]	006820	9
18	*Faucicola boevrei* (Kodjo *et al*. 1997) Sbissi *et al*. 2025	comb. nov. [basonym: *Moraxella boevrei* Kodjo *et al*. 1997]	006820	9
18	*Lwoffella* Sbissi *et al*. 2025	gen. nov.	006820	9
18	*Lwoffella lincolnii* (Vandamme *et al*. 1993) Sbissi *et al*. 2025	comb. nov. [basonym: *Moraxella lincolnii* Vandamme *et al*. 1993]	006820	9
19	*Chryseobacterium gossypii* Mulati *et al*. 2025	sp. nov.	006817	6
20	*Dickeya ananatis* Dobhal *et al*. 2025	sp. nov.	006822	15
21	*Planosporangium spinosum* Tongtoht *et al*. 2025	sp. nov.	006825	7
22	*Cellvibrio chitinivorans* Xin *et al*. 2025	sp. nov.	006827	7
23	*Algoriphagus aurantiacus* Zheng *et al*. 2025	sp. nov.	006826	8
23	*Algoriphagus persicinus* Zheng *et al*. 2025	sp. nov.	006826	10
24	*Dellaglioa kimchii* Ko *et al*. 2025	sp. nov.	006829	7

*The article erroneously states Ang Li Xuang *et al.* 2025. A corrigendum was published [3].

†Not nom. nov. as given by the authors, but sp. nov. Homotypic synonym: *‘Leifsonia flava*’ Cai *et al.* 2018 (effectively but not validly published name).

‡The basonym has a risk group of 2 in the German risk group classification.

The following taxonomic opinions (i.e. the emendation of circumscriptions or the creation of synonyms) do not affect the status of a name under the International Code of Nomenclature of Prokaryotes. These taxonomic opinions can be considered neither as approved nor as disapproved by the International Committee on Systematics of Prokaryotes.

**Table:** 

Name/authors:	Article no.	Page no.
*Antiquaquibacter* Toumi *et al*. 2023 emend. Gtari *et al*. 2025	006809	8
*Faucicola* Humphreys *et al*. 2015 emend. Sbissi *et al*. 2025	006820	9
*Glaciibacter* Katayama *et al*. 2009 emend. Gtari *et al*. 2025	006809	10
*Homoserinimonas* Kim *et al*. 2012 emend. Gtari *et al*. 2025	006809	9
*Moraxella* Lwoff 1939 (Approved Lists 1980) emend. Sbissi *et al*. 2025	006820	8
*Salinibacterium* Han *et al*. 2003 emend. Gtari *et al*. 2025	006809	9
